# Sheep Monoclonal Antibodies Prevent Systemic Effects of Botulinum Neurotoxin A1

**DOI:** 10.3390/toxins4121565

**Published:** 2012-12-19

**Authors:** Jean Mukherjee, Chase McCann, Kwasi Ofori, Julia Hill, Karen Baldwin, Charles B. Shoemaker, Peter Harrison, Saul Tzipori

**Affiliations:** 1 Tufts Cummings School of Veterinary Medicine, North Grafton, MA 01536, USA; 2 Bioventix Limited, Farnham Surrey, UK

**Keywords:** botulinum neurotoxin, botulinum toxin, BoNT, BoNT/A1, BoNT/A2, monoclonal antibodies, sheep monoclonal antibodies, immunotherapy, passive immunization, botulism

## Abstract

Botulinum neurotoxin (BoNT) is responsible for causing botulism, a potentially fatal disease characterized by paralysis of skeletal muscle. Existing specific treatments include polyclonal antisera derived from immunized humans or horses. Both preparations have similar drawbacks, including limited supply, risk of adverse effects and batch to batch variation. Here, we describe a panel of six highly protective sheep monoclonal antibodies (SMAbs) derived from sheep immunized with BoNT/A1 toxoid (SMAbs 2G11, 4F7) or BoNT/A1 heavy chain C-terminus (HcC) (SMAbs 1G4, 5E2, 5F7, 16F9) with or without subsequent challenge immunization with BoNT/A1 toxin. Although each SMAb bound BoNT/A1 toxin, differences in specificity for native and recombinant constituents of BoNT/A1 were observed. Structural differences were suggested by pI (5E2 = 8.2; 2G11 = 7.1; 4F7 = 8.8; 1G4 = 7.4; 5F7 = 8.0; 16F9 = 5.1). SMAb protective efficacy *vs.* 10,000 LD50 BoNT/A1 was evaluated using the mouse lethality assay. Although not protective alone, divalent and trivalent combinations of SMabs, IG4, 5F7 and/or 16F9 were highly protective. Divalent combinations containing 0.5–4 μg/SMAb (1–8 μg total SMAb) were 100% protective against death with only mild signs of botulism observed; relative efficacy of each combination was 1G4 + 5F7 > 1G4 + 16F9 >> 5F7 + 16F9. The trivalent combination of 1G4 + 5F7 + 16F9 at 0.25 μg/SMAb (0.75 μg total SMAb) was 100% protective against clinical signs and death. These results reflect levels of protective potency not reported previously.

## 1. Introduction

Botulinum neurotoxin (BoNT), primarily produced by *Clostridium botulinum*, is responsible for causing botulism in a variety of species, including humans, horses and waterfowl. Botulism is an acute, progressive and potentially fatal disease characterized by flaccid paralysis of skeletal muscle. In humans, botulism classically occurs following ingestion of pre-formed BoNT present within improperly preserved produce; infants may also develop botulism following ingestion of *C. botulinum* spores [1].

There are seven toxinotypes of BoNT, designated A–G. Each BoNT toxinotype is synthesized as a single ~150 kD polypeptide comprised of two subunits linked by a disulfide bond, namely a ~50 kD catalytic light chain (Lc) and a ~100 kD heavy chain (Hc), which is further divided into an N-terminal translocation domain (HcN) and a C-terminal membrane binding domain (HcC) [2,3]. The mechanism of each BoNT toxinotype is similar—following systemic absorption, the Hc facilitates binding and endocytosis of BoNT into motor neurons; within the acidified endosome, the Hc and Lc dissociate; free Lc then binds and hydrolyzes SNARE proteins responsible for docking and release of acetylcholine within the neuromuscular junction [2]. Once endocytosed, BoNT activity is irreversible and can result in death due to flaccid paralysis of muscles associated with respiration.

Due to its potency, ease of production, lack of immunity within the general population, lack of effective specific treatment modalities and ability to induce large-scale fatal effects when ingested or inhaled, there is justified concern that BoNT could be used as a bioterrorist agent via adulteration of food and/or water sources. Consequently, both BoNT and BoNT-producing *Clostridium* sp. are classified as CDC/USDA Select Agents. Patients affected by BoNT require constant, intensive, prolonged supportive care, including maintenance of nutritional and hydration status, personal care, and depending on extent of paralysis, mechanical ventilation [1]. Recovery is dependent upon restoration of neuronal function and appropriate physical therapy [4]. 

Currently, there are no drugs available to prevent or reverse intoxication due to BoNT and although available, immunization is contraindicated due to the increasing use of BoNT as a therapeutic [5,6]. Thus, passive immunotherapy, along with supportive care and mechanical ventilation, are the primary means of treating botulism. Two immunotherapeutic preparations are available, including BIG-IV (BabyBIG), a human IgG preparation licensed for use in infants, and an unlicensed pentavalent polyclonal equine antisera preparation for use in adults [7,8,9]. Both preparations are polyclonal and derived from immunized humans or horses. Thus, (1) supplies are limited; (2) equine antisera carries the risk of serum sickness and anaphylaxis and can only be given once due to development of anti-equine antibodies; (3) human antisera carries the risk of blood-borne disease; and (4) minimizing batch variation to ensure quality and efficacy is difficult. In contrast to polyclonal antisera, monoclonal antibodies (mAbs) can be produced *in vitro*, thereby ensuring an unlimited supply of a highly purified, well-characterized product, devoid of contaminating proteins. The cost-effectiveness of this approach, however, relies on the generation of potent high affinity mAbs. The difficulty, however, has been identifying mAbs that protect against >1000 LD50 when administered at relatively low doses, *i.e.*, <0.5 mg/kg.

To date, several panels of mAbs have been evaluated for protective efficacy against botulinum toxin A1 (BoNT/A1)*in vivo*. Pless *et al.* [10] generated a panel of four mAbs (4A2, 6B2, 6C2, 6E9) via immunization of mice with BoNT/A1 HcC. When administered alone at an unspecified dose, these mAbs provided 100% protection against 10 LD50 BoNT/A1 [10]. Marks *et al.* generated a panel of three mAbs via phage display from mice and humans immunized with BoNT/A HcC + BoNT/A1 (C25, S25) [11] or pentavalent botulinum toxoid (3D12) [12], respectively. When administered at a total dose of 50 µg/mouse (2.5 mg/kg), these mAbs (50 μg mAb/mouse) did not alone prevent death; divalent combinations (25 µg each mAb/mouse) prevented death *vs.* 100–500 LD50 BoNT/A1; and a trivalent combination (S25 + C25 + 3D12; 16.5 µg each mAb/mouse) prevented death *vs.* 10,000 LD50 BoNT/A1 [12]. Cheng *et al.* evaluated the *in vivo* efficacy of two mouse mAbs (F1-2, F1-40), generated via immunization with BoNT/A1 toxoid, *vs.* 143 LD50 BoNT/A1. Protection was achieved when F1-2, F1-40 or F1-2 + F1-40 were administered at total doses of 20, 80 or 8 μg/mouse (4 μg/mAb), respectively [13,14].

Here, we describe the derivation, characterization and *in vivo* efficacy of six sheep monoclonal antibodies (SMAbs) derived from immunization with BoNT/A1 toxoid, HcC or LHn with or without subsequent challenge immunization with BoNT/A1 toxin. Alone, these SMAbs were found to be poorly protective; however, when administered in bi- or tri-valent combinations, selected SMAbs provided 100% survival against 10,000 LD50 BoNT/A1 when administered at doses as low as 0.75 μg/mouse or 0.0375 mg/kg. 

## 2. Results and Discussion

### 2.1. SMAb Derivation and Specificity Patterns

**Figure 1:**
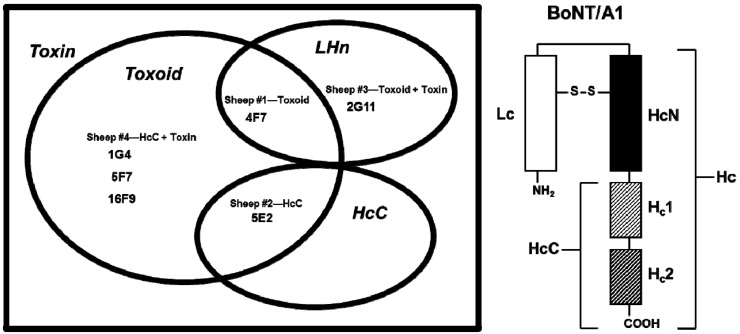
Ven diagram showing derivation and specificity pattern of SMAbs 1G4, 2G11, 4F7, 5E2, and 5F7. Inset shows location of the BoNT/A1 light chain (Lc), heavy chain (Hc), heavy chain N-terminal (HcN) and heavy chain C-terminal (HcC) domains. SMAbs are grouped according to derivation (*i.e.*, sheep number and immunization scheme). BoNT/A1-derived antigens utilized for determining SMAb specificity patterns are italicized and include toxin, toxoid, HcC and LHn (comprising Lc and HcN).

SMAbs 4F7, 5E2 and 2G11 were derived from three sheep immunized with BoNT/A1 toxoid (Sheep #1), BoNT/A1 HcC (Sheep #2) or BoNT/A1 toxoid + toxin (Sheep #3), respectively; SMAbs 1G4, 5F7 and 16F9 were derived from one sheep immunized with BoNT/A1 HcC + BoNT/A1 (Sheep #4). The SMAb specificity pattern for BoNT/A1 toxin, BoNT/A1 toxoid, BoNT/A1 HcC (comprising the C-terminal region of Hc) and LHn (comprising Lc and HcN) was determined via ELISA. All six SMAbs bound BoNT/A1 toxin; however, differences in binding to BoNT/A1 toxoid, BoNT/A1 HcC and LHn were observed. Specifically, 1G4, 5F7 and 16F9 bound BoNT/A1 toxoid; 2G11 bound LHn; 4F7 bound both BoNT/A1 toxoid and LHn; and 5E2 bound BoNT/A1 toxoid and HcC (Figure 1). Based on sequence analysis, these SMAbs are of the IgG1λ isotype (data not shown).

### 2.2. Relative Binding to BoNT/A1 and BoNT/A2

**Figure 2:**
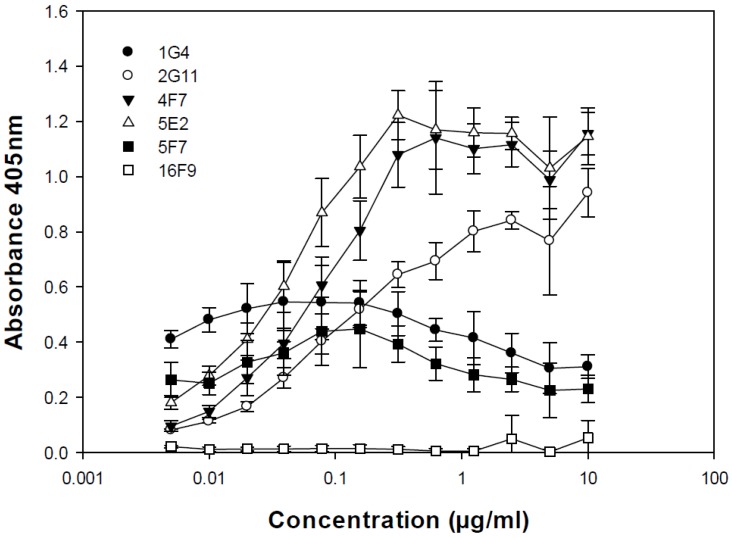
Relative binding of SMAbs to BoNT/A1. Shown is a representative of three replicates.

Relative binding to BoNT/A1 and BoNT/A2 was determined via ELISA. The relative strength of binding to BoNT/A1 at 1 µg/mL was: 5E2 > 4F7 > 2G11 > 1G4 > 5F7 >> 16F9 (Figure 2). Only SMAbs 2G11 and 4F7 exhibited slight cross-reactive binding to BoNT/A2 (A_405_ = 0.4–0.5 at 10 µg/mL; data not shown). 

### 2.3. Specificity for BoNT/A1 Hc and Lc

**Figure 3:**
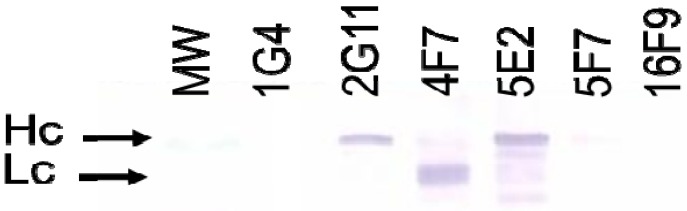
Western blot analysis to evaluate SMAb BoNT/A1 Hc and Lc specificity. Bands corresponding to the Hc and Lc BoNT/A1 were identified based on published molecular weights [15]. Shown is a representative of three replicates.

Western blot analysis was performed to evaluate the specificity of SMAbs 1G4, 2G11, 4F7, 5E2, 5F7 and 16F9 for BoNT/A1 Hc and Lc (Figure 3). Based on the binding patterns revealed, SMAbs 2G11 and 5E2 bind BoNT/A1 Hc and SMAb 4F7 binds BoNT/A1 Lc. No binding was observed for SMAbs 1G4, 5F7 and 16F9, thus specificity for BoNT/A1 Hc and Lc could not be determined. Previous attempts to identify linear protective epitopes of BoNT/A1 suggest these epitopes are easily denatured and may be discontinuous or conformational [10]. Alternatively, SMAbs 1G4, 5F7 and 16F9 may be unable to bind insoluble BoNT/A1.

### 2.4. Sandwich ELISA

**Figure 4:**
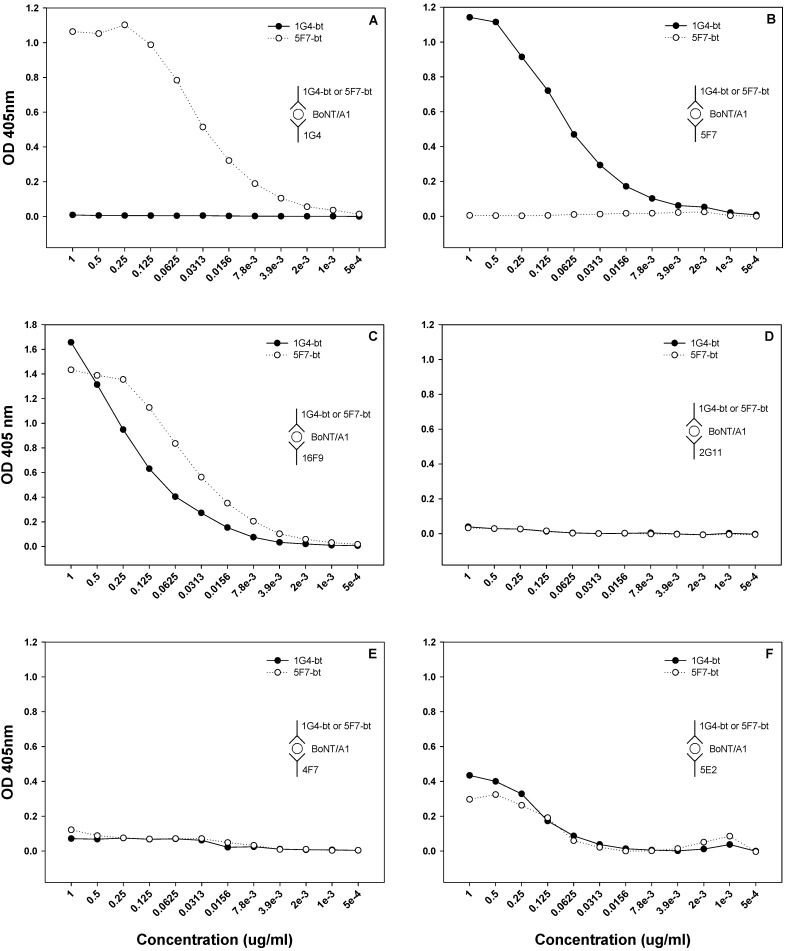
Sandwich ELISAs to determine whether SMAbs 1G4, 2G11, 4F7, 5E2, 5F7 and 16F9 recognized similar or dissimilar epitopes on BoNT/A1. Microtiter plates were coated with 10 μg/mL of SMAb 1G4, 2G11, 4F7, 5E2, 5F7 or 16F9, followed by the addition of 1 μg/mL BoNT/A1. Biotinylated SMAbs 1G4 and 5F7 (1G4-bt and 5F7-bt) were then serially serially diluted 1:2 from 1 to 0.0005 μg/mL. Shown is a representative of two replicates.

A sandwich ELISA was performed to further delineate the relative epitope specificity of SMAbs 1G4, 2G11, 4F7, 5F7 and 16F9 for BoNT/A1 (Figure 4). When SMAbs 1G4 or 5F7 were plate-bound, binding of SMAb 5F7-bt or 1G4-bt, respectively, occurred (Figure 4, Panels A and B); when SMAb 16F9 was plate-bound, SMAb 1G4-bt and 5F7-bt binding occurred (Figure 4, Panel C). Thus, SMAbs 1G4, 5F7, and 16F9 recognize three non-overlapping epitopes within BoNT/A1. When SMAbs 2G11 or 4F7 were plate-bound, no binding of SMAbs 1G4-bt or 5F7-bt occurred (Figure 4, Panels D and E). When SMAb 5E2 was bound, minimal binding of SMAbs 1G4 and 5F7 occurred (Figure 4, Panel F). Thus, SMAbs 2G11, 4F7 and 5E2 may either recognize epitopes, which are similar or adjacent to those recognized by 1G4 and 5F7 or, alternatively, may be unable to bind soluble BoNT/A1.

### 2.5. SMAb Isoelectric Point (pI)

**Figure 5:**
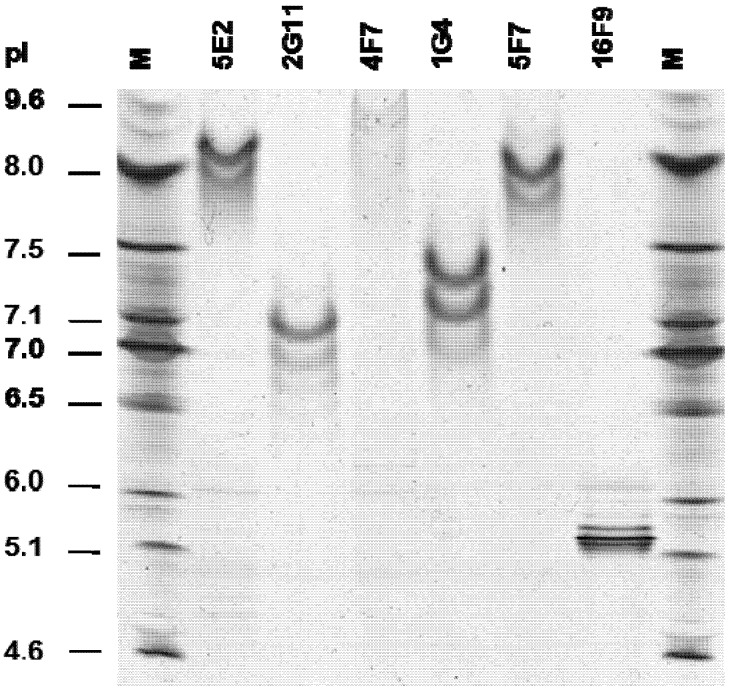
Determination of SMAb pI by IEF. Shown is a representative of two replicates.

The pIs of SMAbs 1G4, 2G11, 4F7, 5E2, 5F7 and 16F9 were determined via isoelectric focusing electrophoresis (IEF) on an IEF gel with a gradient of pH 3–10 (Figure 5). Based on the banding pattern observed relative to the MW marker, the SMAb pIs were: 5E2 = 8.2; 2G11 = 7.1; 4F7 = 8.8; 1G4 = 7.4; 5F7 = 8.0; and 16F9 = 5.1. The differential pIs, along with differences in banding pattern, support the observation that these SMAbs are structurally different.

### 2.6. *In Vivo* Neutralization of BoNT/A1

**Figure 6:**
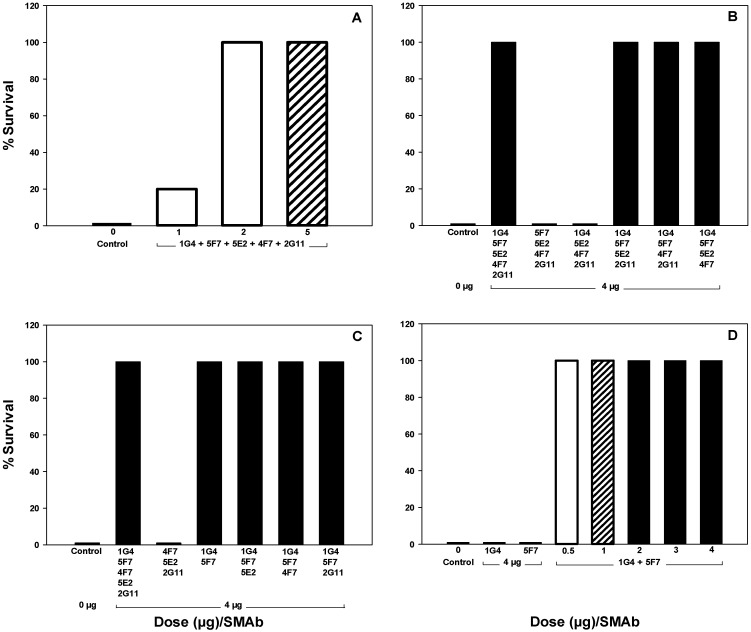
Survival of CD-1 mice given SMAbs 1G4, 2G11, 4F7, 5E2 and/or 5F7 *vs.* 10,000 LD50 BoNT/A1. SMAbs were incubated 30 min. at 25 °C with BoNT/A1 prior to i.p. administration. Panel A—Mice (n = 5) were given combinations of SMAbs 1G4 + 2G11 + 4F7 + 5E2 + 5F7 containing 5, 2, or 1 µg/SMAb (total SMAb dose = 25, 10, or 5 µg, respectively). Panel B—Mice (n = 5) were given combinations containing all five or four of five of the SMAbs 1G4, 2G11, 4F7, 5E2 or 5F7 at a dose of 4 µg/SMAb (total SMAb dose = 20 or 16 µg/mouse, respectively). Panel C—Mice (n = 10) were given combinations containing all five or three of the five SMAbs 1G4, 2G11, 4F7, 5E2 or 5F7 or the divalent combination of SMAbs 1G4 + 5F7 at a dose of 4 µg/SMAb (total SMAb dose = 8–20 µg/mouse). Panel D—Mice (n = 5) were given SMAbs 1G4 or 5F7 alone at a dose of 4 µg or in combination at doses of 0.5–4 µg/SMAb (total SMAb dose = 1–8 µg/mouse).

Using the mouse lethality assay, a series of *in vivo* studies were performed with various combinations of SMAbs 1G4, 2G11, 4F7, 5F7, 5E2 and 16G9. Animals were scored for both survival and development of clinical signs consistent with botulism. An initial study indicated that in combination, at a dose of 10 µg each, SMAbs 1G4 + 2G11 + 4F7 + 5F7 + 5E2 (total SMAb dose = 50 µg/mouse), prevented both clinical signs and death *vs.* 10,000 LD50 BoNT/A1 (data not shown). Thus, a dose-response study was conducted in which the combination of SMAbs 1G4 + 2G11 + 4F7 + 5F7 + 5E2 was administered at doses of 1, 2 and 5 μg each (total SMAb dose = 5, 10 and 25 μg/mouse, respectively). Although mice that received 25 µg total SMAb were fully protected against development of clinical signs and death, those that received 10 μg total SMAb were protected against death, but developed moderate to severe clinical signs of botulism; only 20% of mice that received 5 µg total SMAb survived (Figure 6, Panel A). 

To determine whether all five SMAbs within this combination were necessary for protective efficacy, two studies were conducted in which one or two SMAbs were eliminated from the combination. For each of these studies, a dose of 4 μg of each SMAb was used *vs.* 10,000 LD50 BoNT/A1. The first study involved evaluating the effect of eliminating a single SMAb. Mice that received either all five SMAbs (1G4 + 2G11 + 4F7 + 5F7 + 5E2; total SMAb dose = 20 µg) or a combination of four SMAbs that included both 1G4 and 5F7 along with any two additional SMAbs were fully protected against development of both clinical signs and death. In contrast, mice which received a combination lacking both 1G4 and 5F7 did not survive, indicating that both 1G4 and 5F7 were necessary for protective efficacy (Figure 6, Panel B). The second study involved evaluating the effect of eliminating two SMAbs. Mice that received 1G4 + 5F7 +/- any additional SMAb were fully protected against development of both clinical signs and death. In contrast, mice that received the combination of 2G11 + 4F7 + 5E2 did not survive. This study, therefore, demonstrated that SMAbs 1G4 and 5F7 in combination were both necessary and sufficient for protective efficacy (Figure 6, Panel C). 

To determine the minimum dose of 1G4 and 5F7 required for protective efficacy, a dose-response study was conducted in which SMAbs 1G4 and 5F7 were administered at doses of 0, 0.5, 1, 2, 3 and 4 μg each (total SMAb dose = 0, 1, 2, 4, 6 and 8 µg/mouse, respectively). Mice that received a minimum of 2 μg of each SMAb (total SMAb dose = 4 µg) were fully protected against development of clinical signs and death. However, those that received 1 or 0.5 μg of each SMAb (total SMAb dose = 2 or 1 μg/mouse, respectively) survived, but developed mild or moderate-severe signs of botulism, respectively (Figure 6, Panel D).

**Figure 7:**
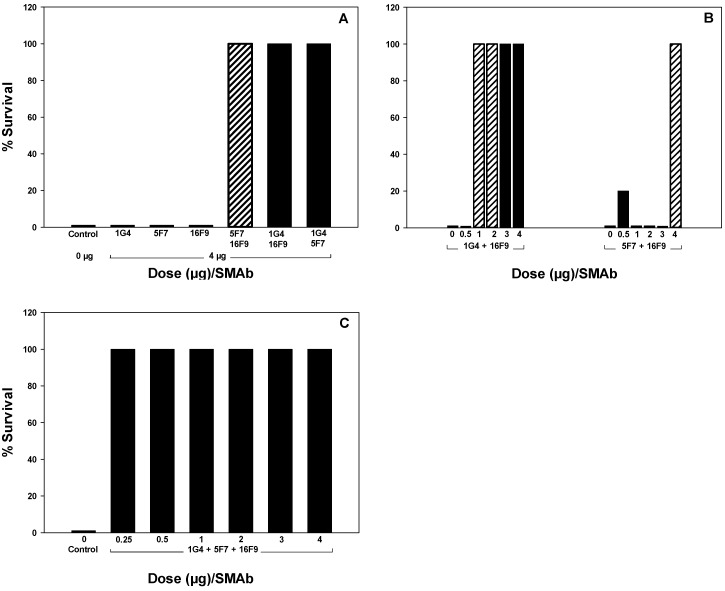
Survival of CD-1 mice given combinations containing SMAbs 1G4, 5F7 and/or 16F9 *vs.* 10,000 LD50 BoNT/A1. SMAbs were incubated 30 min. at 25 °C with BoNT/A1 prior to i.p. administration. Panel A–Mice (n = 5) were given SMAbs 1G4, 5F7 or 16F9 either alone or as divalent combinations at doses of 4 µg/SMAb (total SMAb dose = 4–8 µg/mouse). Panel B—Mice (n = 5) were given divalent combinations of SMAbs 1G4 + 16F9 or 5F7 + 16F9 at doses of 0.5–4 µg/SMAb (total SMAb dose = 1–8 µg/mouse). Panel C—Mice (n = 5) were given trivalent combinations of SMAbs 1G4 + 5F7 +16F9 at doses of 0.25, 0.5, 1, 2, 3 or 4 µg/SMAb (total SMAb dose = 0.75, 1.5, 3, 6, 9 or 12 µg/mouse).

A series of studies was conducted to evaluate the relative efficacy of SMAb combinations containing SMAb 16F9, which, similar to SMAbs 1G4 and 5F7, binds BoNT/A1 toxin and toxoid. The first study was performed to determine whether SMAb 16F9 could substitute for either SMAb 1G4 or 5F7. This study utilized a dose of 0 or 4 µg of each SMAb (total SMAb dose = 4 or 8 μg/mouse) *vs.* 10,000 LD50 BoNT/A1. Mice that received 1G4, 5F7 or 16F9 alone did not survive, whereas mice that received 1G4 + 5F7, 1G4 + 16F9 or 5F7 + 16F9 were fully protected against death due to BoNT/A1. These results indicate that 16F9 can substitute for either 1G4 or 5F7 (Figure 7, Panel A). 

Dose-response studies utilizing divalent and trivalent combinations of SMAbs 1G4, 5F7 and 16F9 were then performed. To evaluate divalent combinations, SMAbs 1G4 or 5F7 were administered in combination with SMAb 16F9 at doses of 0, 0.5, 1, 2, 3 or 4 µg each (total SMAb dose = 4, 1, 2, 4, 6 or 8 μg/mouse, respectively). Mice which received 1G4 + 16F9 at doses of 1–4 µg each (total SMAb dose = 2–8 μg/mouse, respectively) were protected against death, but those which received ≤2 µg of each SMAb developed mild signs of botulism. Mice which received 5F7 + 16F9 at a dose of 4 μg each (total SMAb dose = 8 µg/mouse) were fully protected against death but developed mild signs of botulism; with the exception of one outlier given 0.5 µg/SMAb, mice that received 0.5–3 μg of SMAb 5F7 and 16F9 each (total SMAb dose = 1–6 µg/mouse) did not survive. Based on the clinical signs and mortality observed of mice receiving 1 µg/SMAb, the relative efficacy of divalent combinations of 1G4, 5F7 and 16F9 is: 1G4 + 16F9 ≈ 1G4 + 5F7 >> 5F7 + 16F9 (Figure 6, Panel D and Figure 7, Panel B). To evaluate trivalent combinations, SMAbs 1G4, 5F7 and 16F9 were administered at doses of 0.25, 0.5, 1, 2, 3 and 4 μg each (total SMAb dose = 0.75, 1.5, 3, 6, 9 and 12 µg/mouse, respectively). Mice thta received doses of 0.25–4 µg of each SMAb (total SMAb dose = 0.75–12 μg/mouse) were protected against development of both clinical signs and death, indicating that the trivalent combination of SMAbs 1G4, 5F7 and 16F9 is more effective than divalent combinations of these SMAbs (Figure 7, Panel C).

## 3. Experimental Section

### 3.1. Botulinum Neurotoxin and Immunogens

Botulinum neurotoxin A1 (BoNT/A1), botulinum neurotoxin A2 (BoNT/A2) and BoNT/A1 toxoid were purchased from Metabiologics, Inc. (Madison, WI). All work with BoNT was performed in a CDC-registered select agent facility. BoNT/A1 heavy chain C-terminus (BoNT/A1 HcC) was isolated, purified, and quantitated as described previously [16]. LHn, a recombinant entity comprised of Lc and HcN, was provided by the Health Protection Agency (Porton Down, UK) [3].

### 3.2. Sheep Immunization

Sheep were immunized multiple times with 50–250 µg BoNT/A1 toxoid (Sheep #1 and #3) or BoNT/A1 HcC (Sheep #2 and #4), emulsified at a 1:1 v/v ratio in Freund’s Complete Adjuvant (initial immunization only; Pierce Biotechnology, Rockford, IL), Alum or Freund’s Incomplete Adjuvant (all subsequent immunizations; Pierce Biotechnology, Rockford, IL) ± 600 μg CpG (Coley Pharmaceuticals #2395, Worcester, MA) administered at ≥3-week intervals in multiple sites within the area surrounding the left and right axillary and/or superficial cervical lymph nodes either intramuscularly (four sites in axillary and shoulder region; Sheep #1 and 2) or subcutaneously (four–eight sites within pre-scapular region; Sheep #3 and 4). Sheep #3 and 4 were then boosted with increasing doses of BoNT/A1 (up to 4 µg/sheep). BoNT/A1 was administered in 3 mL phosphate buffered saline (PBS) every three–four days subcutaneously in multiple sites surrounding the superficial cervical lymph node. 

Titers were evaluated periodically via ELISA. Briefly, microtiter plates (Costar #9018, Corning, NY or Nunc Immobilon or Nunc Immuno-Maxisorp, Rochester, NY) were coated with 1 µg/µL BoNT/A1 toxin (Sheep #3 and 4), BoNT/A1 toxoid (Sheep #1) or HcC (Sheep #2) and blocked with 1% bovine serum albumin (BSA; ICN Pharmaceuticals, Costa Mesa, CA) in PBS. Serum samples were serially diluted 1:2. Assays were developed using alkaline phosphatase (AP)-labeled rabbit anti-sheep IgG (Southern Biotechnology Associates #6150-04, Birmingham, AL) or donkey anti-sheep IgG (Sigma-Aldrich #A5187, St. Louis, MO) followed by the addition of 1 mg/mL *p-*nitrophenyl phosphate (Sigma-Aldrich, St. Louis, MO). Absorbance at 405 nm (A_405_) was determined. Titer was defined as the greatest dilution that gave an O.D. ≥ 2.5× the background. 

### 3.3. Sheep Monoclonal Antibodies (SMAbs)

SMAbs were generated via standard methods [17] using lymphocytes isolated from the axillary (Sheep #1 and #2) or superficial cervical (Sheep #3 and 4) lymph nodes. Briefly, lymph nodes were removed and crushed to release lymphocytes into Dulbecco’s Modified Eagle’s Medium (DMEM) + 1% Penicillin/Streptomycin. The cell suspension was pelleted via centrifugation at 1200 rpm for 10 minutes to isolate the lymphocytes. Isolated lymphocytes were fused to 1C10, a proprietary, non-productive, sheep heteromyeloma, produced by inter-species back-fusion to the mouse myeloma NS1 (Bioventix, Ltd., Farnham Surrey, UK) by standard methods [17]. Supernatants from hypoxanthine-aminopterin-thymidine (HAT)-selected hybridomas were successively screened by ELISA on microtiter plates coated with 1 µg/mL BoNT/A1 and developed with AP-labeled rabbit anti-sheep IgG (Southern Biotechnology Associates #6150-04, Birmingham, AL) or donkey anti-sheep IgG (Sigma-Aldrich #A5187, St. Louis, MO). Stable, positive clones were selected by subconing at least twice by soft-agar cloning [17,18]. A total of six SMAbs, derived from lymphocytes isolated from the superficial cervical lymph nodes of Sheep #1-4, were selected for further study. Purified SMAb was isolated from the corresponding hybridomas culture supernatants via Protein A chromatography using a Prosep A column (Millipore, Billerica, MA). Purified SMAbs were quantitated via A_280_.

### 3.4. Relative Binding and Specificity of SMAbs by ELISA

The relative binding of each SMAb to BoNT/A1 and BoNT/A2 was determined by ELISA. Briefly, microtiter plates (Costar #9018, Corning, NY) were coated with 1 µg/mL BoNT/A1 or BoNT/A2 and blocked with 1% bovine serum albumin (BSA; ICN Pharmaceuticals, Costa Mesa, CA) in PBS. Purified SMAbs were serially diluted 1:2 in triplicate across plate from 10 to 0.005 µg/mL. The assay was developed using AP-labeled rabbit anti-sheep IgG (Southern Biotechnology Associates #6150-04, Birmingham, AL), followed by the addition of 1 mg/mL *p-*nitrophenyl phosphate (Sigma-Aldrich #S0942, St. Louis, MO). A_405_ was determined and plotted *vs.* concentration. 

The relative specificity of each SMAb for BoNT/A1 toxoid, LHn and HcC was determined by ELISA. Microtiter plates (Nunc, Rochester, NY) were coated with 0.2–1 μg/mL BoNT/A1 toxoid, LHn or HcC and blocked with reconstituted nonfat dry milk. Following incubation with hybridoma culture supernatants, the assay was developed using AP-labeled rabbit anti-sheep IgG (Southern Biotechnology Associates #6150-04, Birmingham, AL) or donkey anti-sheep IgG (Sigma-Aldrich #A5187, St. Louis, MO), followed by the addition of 1 mg/mL *p-*nitrophenyl phosphate (Sigma-Aldrich #S0942, St. Louis, MO). A_405_ was determined and plotted *vs.* concentration. 

### 3.5. Western Blot Analysis of SMAbs

The subunit specificity of each SMAb was evaluated by Western blot. BoNT/A1 was electrophoresed by SDS-PAGE on a 12%, 10 x 14 cm acrylamide slab gel under reducing conditions and then electrophoretically transferred to a 0.2 µm nitrocellulose membrane (BioRad, Hercules, CA). Following electrophoretic transfer, the membrane was soaked 3 h in BLOTTO (2% non-fat milk + 5 mM NaN_3_ + TBS-T). A 25 lane miniblotter apparatus (Immunetics, Boston, MA) was used to divide the membrane into discrete lanes. The membrane was washed five times (three times with TBS + 0.1% Tween-20, then twice with BLOTTO) between each of the following steps. SMAbs were incubated at 10 µg/mL in BLOTTO in individual lanes in the presence of membrane-bound BoNT/A1 for 2–3 h at room temperature. A 1:1500 dilution of AP-labeled rabbit anti-sheep IgG (Southern Biotechnology Associates #6150-04, Birmingham, AL) was added to each lane. The blot was developed using Sigma-Aldrich Fast™ BCIP/NBT substrate (Sigma-Aldrich, St. Louis, MO). 

### 3.6. Sandwich ELISA

Sandwich ELISAs were performed to determine whether SMAbs 1G4, 2G11, 4F7, 5E2, 5F7 and 16F9 recognized similar or different epitopes on BoNT/A1. This ELISA was performed by coating microtiter plates with an SMAb, which was used to capture BoNT/A1. Biotinylated detection SMAbs (1G4-bt or 5F7-bt) were then serially diluted across the microtiter plate. 

Briefly, microtiter plates (Costar #9018, Corning, NY) were coated with 50 μL/well of a 10 μg/mL solution of SMAb 1G4, 2G11, 4F7, 5E2, 5F7 or 16F9. Plates were blocked with 1% BSA (ICN Pharmaceuticals, Costa Mesa, CA) in PBS. Following coating, 50 μL of 1 μg/mL BoNT/A1 in 1% BSA + PBS was added to each well. Plates were incubated and washed. The detection SMAbs, 1G4-bt or 5F7-bt, were serially diluted 1:2 in triplicate from 1 to 0.0005 μg/mL across each plate. The assay was developed using a 1:1000 dilution of AP-labeled streptavidin (Sigma-Aldrich Co., St. Louis, MO), followed by addition of 1 mg/mL p-nitrophenyl phosphate (Sigma-Aldrich Co., St. Louis, MO). A_405_ was determined. 

### 3.7. SMAb Isoelectric Point

The pIs of SMAbs 1G4, 2G11, 4F7, 5E2, 5F7 and 16F9 were determined via isoelectric focusing (IEF). 5 μg of each SMAb was diluted 1:1 with Novex IEF, pH 3–10 sample buffer (Invitrogen #LC5311, Carlsbad, CA) in a total volume of 10 μL and loaded onto a Novex IEF gel (Invitrogen, Carlsbad, CA), pH 3–10 *vs.* an IEF marker (BioRad #161-0310, Hercules, CA). Gel was run using a XCell II SureLock Mini-Cell System (Invitrogen, Carlsbad, CA) with 1× IEF cathode (upper chamber) and anode (lower chamber) buffers (Invitrogen #LC5310 and #LC5300, respectively, Carlsbad, CA). Gel was run for 1 h at 100 V, followed by 1 h at 200 V, then 30 min. at 500 V. The gel was fixed for 30 min. in 12% TCA containing 3.5% sulfosalicylic acid and then stained with Coomassie blue (Invitrogen #LC6025, Carlsbad, CA). The pI of each SMAb was determined using a standard curve generated based on the migration pattern of proteins within the marker.

### 3.8. Murine Lethality Assay

The murine lethality assay was used to examine the ability of SMAbs either alone or in combination to neutralize the effects of BoNT/A1 *in vivo*. These studies were conducted at Tufts Cummings School of Veterinary Medicine under an approved animal care and use protocol. Groups of 5–10 female CD-1 mice ~20 g each (Charles River, Wilmington, MA) were used. One day prior to each study, mice were weighed and sorted to reduce inter-group weight variation. BoNT/A1 was dosed on a pg/g basis at 10,000 LD50 (calculated based on the LD50 dose determined via conventional mouse lethality assay; for the BoNT/A1 utilized in these studies, 1 LD50 = 0.9 pg/g mouse weight) using the average weight of all mice included within each experimental study. Combinations of SMAbs 1G4, 2G11, 4F7, 5E2, 5F7 and 16F9 at combined doses of 0–50 μg/mouse were evaluated. SMAb combinations were pre-incubated with BoNT/A1 in PBS containing 0.2% gelatin (Sigma-Aldrich #G0411, St. Louis, MO) for 30 min. prior to i.p. administration in a 200 μL volume/mouse. Mice were observed 2–6×/day for survival and development of clinical signs of botulism, including severity of abdominal breathing, lethargy, decreased ambulation and open-mouth breathing. 

## 4. Conclusions

The development and functional characterization of six BoNT/A1-specific IgG SMAbs is described. These SMAbs were isolated from sheep immunized with four different combinations of BoNT/A1-derived antigens: (1) Toxoid (Sheep #1; SMAb 4F7); (2) HcC (Sheep #2, SMAb 5E2); (3) Toxoid + Toxin (Sheep #3; SMAb 2G11); or (4) HcC + Toxin (Sheep #4; SMAbs 1G4, 5F7, and 16F9). Based on differential binding to BoNT/A1 derivatives, including toxoid, HcC and LHn, each SMAb was segregated into one of four antigen-specificity groups defined by binding BoNT/A1 plus: Toxoid (SMAbs 1G4, 5F7, 16F9); Toxoid + LHn (SMAb 4F7); Toxoid + HcC (SMAb 5E2); or LHn alone (SMAb 2G11). Each antigen-specificity group correlated with immunization with a different set of BoNT/A1-derived antigens. 

Using a murine model of botulism, combinations of SMAbs 1G4, 2G11, 4F7, 5E2, 5F7 and 16F9 were evaluated for the ability to neutralize BoNT/A1 *in vivo*. All *in vivo* studies were performed at a dose of 10,000 LD50 BoNT/A1. Mice were scored for both survival and development of clinical signs. Absence of clinical signs and death were indicative of 100% protection; presence of clinical signs in the absence of death was indicative of partial protection. 

Of the six SMAbs evaluated, only 1G4, 5F7 and 16F9 were found to contribute toward protective efficacy. Interestingly, SMAbs 1G4, 5F7 and 16F9 exhibited little or no binding to insoluble forms of BoNT/A1. In contrast, SMAbs 2G11, 4F7 and 5E2 appeared to bind plate-bound BoNT/A1 quite well, but possibly were unable to bind soluble BoNT/A1. Thus, the relative ability of 1G4, 5F7 and 16F9 *vs.* 2G11, 4F7 and 5E2 to contribute or not contribute to the protective efficacy *in vivo* may be related to the ability or inability, respectively, to bind soluble BoNT/A1, which would be the form present *in vivo*.

Similar to the findings of Nowakowski *et al.* [12] and Cheng *et al.* [13], these SMAbs were only highly protective when administered as divalent or trivalent combinations. The trivalent combination of SMAbs 1G4 + 5F7 + 16F9 provided 100% protection against both clinical signs and death when administered at a dose of 0.25 μg/SMAb/mouse (total SMAb dose = 0.75 μg/mouse) *vs.* 10,000 LD50 BoNT/A1. In contrast, although divalent combinations comprised of 1G4 +5F7 or 1G4 +16F9 at a dose of 1 μg/SMAb/mouse (total SMAb dose = 2 μg/mouse) *vs.* 10,000 LD50 BoNT/A1 provided 100% protection against death, mild clinical signs were observed. 

Relative potencies of the divalent and trivalent SMAb preparations can be calculated in a manner similar to that presented by Nowakowski *et al.* [12], given that one international unit (IU) of antitoxin has been defined as the quantity required to neutralize 10,000 LD50 of BoNT [19]. Based on the minimum dose of each preparation required to prevent death and clinical signs in 100% of mice, the relative potencies are: 1G4 + 5F7 +16F9 (0.25 μg/SMAb/mouse; total SMAb/mouse = 0.75 μg; potency = 1333 IU/mg antitoxin) >>> 1G4 + 5F7 (2 μg/SMAb/mouse; total SMAb/mouse = 4 μg; potency = 250 IU/mg antitoxin) > 1G4 + 16F9 (3 μg/SMAb/mouse; total SMAb/mouse = 6 μg; potency = 166 IU/mg antitoxin) >> 5F7 + 16F9—the potency of this preparation is <125 IU/mg antitoxin, since even at a dose of 4 μg/SMAb/mouse (8 μg total SMAb/mouse), mild clinical signs were observed. The previously described trivalent combination of mAbs C25 + 3D12 + S25 has a potency of 45 IU/mg antitoxin [12]. The potency of the divalent combination of mAbs F1-2 and F1-40 has not been determined [13]. Thus, both the divalent and trivalent combinations of 1G4, 5F7 and 16F9 appear to provide superior efficacy relative to the divalent and trivalent combinations of C25, 3D12 and S25 [12] and the divalent combination of F1-2 and F1-40 [13]. 

Similar to the mouse mAbs C25 and S25 generated by Amersdorfer *et al.* [11], SMAbs 1G4, 5F7 and 16F9 were derived from animals initially immunized with HcC, followed by challenge immunization with lethal doses of BoNT/A1 [11]. This immunization regimen appears to favor isolation of highly neutralizing mAbs. Challenge immunization with BoNT/A1 not only ensures the presence of protective antibodies *in vivo*, but also presumably boosts the response against epitopes present on intact BoNT/A1. Interestingly, in constrast to the results of Pless *et al.* [10], immunization with HcC alone did not result in isolation of protective SMAbs. Similarly, immunization with BoNT/A1 toxoid +/- challenge immunization with BoNT/A1 toxin also did not result in generation of protective SMAbs. Derivation of SMAbs 1G4, 5F7 and 16F9 from sheep as opposed to mice or humans may have provided an intrinsic advantage over previously described BoNT/A1 mAbs [12,13] as ruminants are known to produce higher affinity antibodies than other species [17]. In addition, as has been demonstrated for mouse mAbs C25, S25 and 3D12 [12], it is likely that the divalent and trivalent combinations of SMAbs 1G4, 5F7 and 16F9 are of higher affinity than the individual SMAbs. Determination of SMAb affinity is the focus of ongoing studies. 

Epitope specificity appears to determine protective efficacy and is associated with recognition of epitopes common to BoNT/A1 toxin and toxoid. These protective epitopes are defined by SMAbs 1G4, 5F7 and 16F9. SMAbs 1G4, 5F7 and 16F9 were derived from the same sheep and belong to the same antigen-specificity group; however, these SMAbs did not compete with one another for binding to BoNT/A1, indicating recognition of three separate epitopes common to both BoNT/A1 toxin and toxoid. In contrast, SMAbs 2G11, 4F7 and 5E2, which recognize epitopes present on BoNT/A1 toxin +/- toxoid, and HcC or LHn are not protective. Based on differences in antigen specificity, recognition of Hc *vs.* Lc and differences in competition for binding to BoNT/A1, SMAbs 1G4, 2G11, 4F7, 5E2, 5F7 and 16F9 appear to bind separate but overlapping epitopes within BoNT/A1. Interference with the 1G4 and 5F7 epitopes by SMAbs 2G11, 4F7 and 5E2 may explain why the combination of SMAbs 1G4 + 5F7 + 4F7 + 2G11 + 5E2 at a dose of 10 μg/SMAb appeared to be less protective (mild clinical signs observed) than the combination of 1G4 and 5F7 at 2 μg/SMAb (no clinical signs observed). 

Similar to studies utilizing mAbs C25, S25 and 3D12, recognition of a single epitope defined by SMAbs 1G4, 5F7 or 16F9 was not sufficient; instead, protective efficacy relied on recognition of two or more protective epitopes [12]. We were unable to determine Hc *vs.* Lc binding specifity of SMabs 1G4, 5F7 and 16F9. Neutralizing antibodies such as SMAbs 1G4, 5F7 and 16F9 that lack the ability to bind BoNT within the format of a Western blot have previously been described [20,21]. It has been hypothesized that lack of Hc or Lc recognition may be the result of binding conformational epitopes that are destroyed via SDS denaturation and/or are associated with regions at or near the Hc-Lc junction [21]. Given SMAbs 1G4, 5F7 and 16F9 belong to the same antigen specificity group, the epitopes recognized by these SMAbs are likely within a similar region. Recognition of adjacent or overlapping epitopes on the Hc has been hypothesized to provide increased blockade of the Hc receptor binding region [12,22].

The availability of protective SMAbs provides the opportunity for immunotherapy of individuals exposed to BoNT and prevention of subsequent development of botulism. A single dose of a protective divalent or trivalent SMAb preparation administered soon after exposure would be sufficient to prevent morbidity and/or mortality due to BoNT. SMAb administration is expected to be associated with adverse effects similar to that of equine antisera, including serum sickness and anaphylaxis. In addition, it is anticipated that induction of anti-sheep antibodies would limit use to once per lifetime. Unlike equine antisera, however, the SMAbs can be chimerized to produce a product that is unlikely to induce such adverse effects and that can be administered more than once. Thus, these SMAbs provide an unlimited, potentially safer alternative to both the currently available human and equine polyclonal BoNT therapeutics.
